# RBM46 is essential for gametogenesis and functions in post-transcriptional roles affecting meiotic cohesin subunits

**DOI:** 10.1093/procel/pwac040

**Published:** 2022-09-14

**Authors:** Yue Lv, Gang Lu, Yuling Cai, Ruibao Su, Liang Liang, Xin Wang, Wenyu Mu, Xiuqing He, Tao Huang, Jinlong Ma, Yueran Zhao, Zi-Jiang Chen, Yuanchao Xue, Hongbin Liu, Wai-Yee Chan

**Affiliations:** Shandong Key Laboratory of Reproductive Medicine, Shandong Provincial Hospital Affiliated to Shandong First Medical University, Jinan 250012, China; Center for Reproductive Medicine, Shandong University, Jinan 250012, China; CUHK-SDU Joint Laboratory on Reproductive Genetics, School of Biomedical Sciences, Faculty of Medicine, The Chinese University of Hong Kong, Hong Kong, China; Key Laboratory of Reproductive Endocrinology of Ministry of Education, Shandong University, Jinan 250012, China; CUHK-SDU Joint Laboratory on Reproductive Genetics, School of Biomedical Sciences, Faculty of Medicine, The Chinese University of Hong Kong, Hong Kong, China; Center for Reproductive Medicine, Shandong University, Jinan 250012, China; Key Laboratory of Reproductive Endocrinology of Ministry of Education, Shandong University, Jinan 250012, China; Key Laboratory of RNA Biology, Institute of Biophysics, Chinese Academy of Sciences, Beijing 100101, China; Fertility Preservation Lab, Reproductive Medicine Center, Guangdong Second Provincial General Hospital, Guangzhou 510062, China; Key Laboratory of RNA Biology, Institute of Biophysics, Chinese Academy of Sciences, Beijing 100101, China; Key Laboratory of Systems Health Science of Zhejiang Province, School of Life Science, Hangzhou Institute for Advanced Study, University of Chinese Academy of Sciences, Hangzhou 310024, China; Center for Reproductive Medicine, Shandong University, Jinan 250012, China; Key Laboratory of Reproductive Endocrinology of Ministry of Education, Shandong University, Jinan 250012, China; Center for Reproductive Medicine, Shandong University, Jinan 250012, China; Key Laboratory of Reproductive Endocrinology of Ministry of Education, Shandong University, Jinan 250012, China; Center for Reproductive Medicine, Shandong University, Jinan 250012, China; Key Laboratory of Reproductive Endocrinology of Ministry of Education, Shandong University, Jinan 250012, China; Center for Reproductive Medicine, Shandong University, Jinan 250012, China; CUHK-SDU Joint Laboratory on Reproductive Genetics, School of Biomedical Sciences, Faculty of Medicine, The Chinese University of Hong Kong, Hong Kong, China; Key Laboratory of Reproductive Endocrinology of Ministry of Education, Shandong University, Jinan 250012, China; Shandong Key Laboratory of Reproductive Medicine, Shandong Provincial Hospital Affiliated to Shandong First Medical University, Jinan 250012, China; Center for Reproductive Medicine, Shandong University, Jinan 250012, China; Key Laboratory of Reproductive Endocrinology of Ministry of Education, Shandong University, Jinan 250012, China; Shandong Key Laboratory of Reproductive Medicine, Shandong Provincial Hospital Affiliated to Shandong First Medical University, Jinan 250012, China; Center for Reproductive Medicine, Shandong University, Jinan 250012, China; Key Laboratory of Reproductive Endocrinology of Ministry of Education, Shandong University, Jinan 250012, China; Shanghai Key Laboratory for Assisted Reproduction and Reproductive Genetics, Shanghai 200135, China; Center for Reproductive Medicine, Ren Ji Hospital, School of Medicine, Shanghai Jiao Tong University, Shanghai 200135, China; Key Laboratory of RNA Biology, Institute of Biophysics, Chinese Academy of Sciences, Beijing 100101, China; Center for Reproductive Medicine, Shandong University, Jinan 250012, China; CUHK-SDU Joint Laboratory on Reproductive Genetics, School of Biomedical Sciences, Faculty of Medicine, The Chinese University of Hong Kong, Hong Kong, China; Key Laboratory of Reproductive Endocrinology of Ministry of Education, Shandong University, Jinan 250012, China; CUHK-SDU Joint Laboratory on Reproductive Genetics, School of Biomedical Sciences, Faculty of Medicine, The Chinese University of Hong Kong, Hong Kong, China

**Keywords:** RBM46, LACE-seq, RNA-binding protein, meiosis, cohesin

## Abstract

RBM46 is a germ cell-specific RNA-binding protein required for gametogenesis, but the targets and molecular functions of RBM46 remain unknown. Here, we demonstrate that RBM46 binds at specific motifs in the 3ʹUTRs of mRNAs encoding multiple meiotic cohesin subunits and show that RBM46 is required for normal synaptonemal complex formation during meiosis initiation. Using a recently reported, high-resolution technique known as LACE-seq and working with low-input cells, we profiled the targets of RBM46 at single-nucleotide resolution in leptotene and zygotene stage gametes. We found that RBM46 preferentially binds target mRNAs containing GCCUAU/GUUCGA motifs in their 3ʹUTRs regions. In *Rbm46* knockout mice, the RBM46-target cohesin subunits displayed unaltered mRNA levels but had reduced translation, resulting in the failed assembly of axial elements, synapsis disruption, and meiotic arrest. Our study thus provides mechanistic insights into the molecular functions of RBM46 in gametogenesis and illustrates the power of LACE-seq for investigations of RNA-binding protein functions when working with low-abundance input materials.

## Introduction

The RNA-binding motif protein (RBM) family is a subgroup of RNA-binding proteins (RBPs). RBMs contain multiple RNA-binding domains, among which RNA recognition motifs are the most common (Qin *et al.*, 2020). Like other RBPs, RBM proteins are known to function in various RNA processes, including pre-mRNA splicing, RNA stability, mRNA translation, and RNA editing, thereby influencing multiple physiological processes (Li *et al.*, 2021). As one example, RBM10 is a splicing factor responsible for SAT1 exon 4 skipping. Further, as it prevents SAT1 splicing changes in infected cells, RBM10 can limit viral replication (Pozzi *et al.*, 2020). Studies of the RBM24 protein have shown that it modulates hepatitis B virus replication by targeting the viral RNA and inhibits core protein translation by targeting the terminal redundancy sequence (Yao *et al.*, 2018). RBM47 is known to function in a variety of biological processes by regulating alternative splicing and RNA stability: it can inhibit breast cancer progression by stabilizing tumor suppressor mRNAs (Vanharanta *et al.*, 2014) and has been shown to regulate multiple splicing events in nasopharyngeal carcinoma based on binding with another RBP-hnRNPM (Xu *et al.*, 2021).

The term “cohesin” describes a variety of multisubunit complexes that are essential for sister chromatid cohesion. Cohesin complexes form ring structures to hold sister chromatids together, and this occurs in both mitosis and meiosis. However, there are both compositional and functional distinctions for cohesin complexes between meiosis and mitosis (Klein *et al.*, 1999; Haering and Jessberger, 2012): five universal cohesin subunits (SMC1α, SMC3, RAD21, SA1/SA2) are required for proper cohesin complex function in both meiosis and mitosis (Eijpe *et al.*, 2000; Xu *et al.*, 2004). In mammalian meiosis, there are four meiosis-specific cohesin subunits, including one SMC protein (SMC1β), two kleisins (RAD21L and REC8), and a stromal antigen protein (STAG3) (Prieto *et al.*, 2001; Revenkova *et al.*, 2001; Lee *et al.*, 2003; Lee and Hirano, 2011). The differential combination of specific cohesin subunits enables the formation of at least six cohesin complexes (Jessberger, 2011; Haering and Jessberger, 2012). In meiosis, cohesin complexes load onto sister chromatids during the premeiotic S phase and early meiosis I (leptotene stage) (McNicoll *et al.*, 2013; Biswas *et al.*, 2016), and studies have demonstrated how the meiosis-specific subunits confer specific functions in meiosis-related chromosomal events such as assembly of chromosome axial elements (AEs), DNA double-strand breaks (DSBs), and chromosome synapsis (Xu *et al.*, 2005; Adelfalk *et al.*, 2009; Herran *et al.*, 2011; Llano *et al.*, 2012; Fukuda *et al.*, 2014; Ward *et al.*, 2016).

*Rbm46* is conserved among jawed vertebrates. In mice and most animals, its gene product comprises three RNA recognition motif domains and one C-terminal double-stranded RNA-binding motif (DSRM) domain (Fig. S1A). Microarray-based profiling study of 9- to 18-week human fetal ovaries revealed an increasing trend in *RBM46* expression that steadily increases with gestational age (Houmard *et al.*, 2009; Gerstberger *et al.*, 2014). Single-cell RNA-sequencing analysis of juvenile and aged cynomolgus monkey ovaries found that RBM46 is highly expressed in oocytes of early follicle stages (Wang *et al.*, 2020b). Dai *et al.*, (2021) used TALENs to generate two *rbm46* mutant zebrafish lines and reported that both *rbm46* homozygous mutant zebrafish lines are infertile, failed to initiate meiosis, and did not produce any sperm, concluding that RBM46 may be involved in post-transcriptional regulation during spermatogenesis (Dai *et al.*, 2021). However, these studies have not investigated the *in vivo* binding targets of RBM46. In this study, we performed high-resolution linear amplification of complementary DNA ends and sequencing (LACE-seq) to identify the targets of RBM46 in mice leptotene and zygotene spermatocytes. We show that RBM46 binding at specific motifs in the 3ʹUTRs of mRNAs encoding four meiosis-specific cohesin complex subunits that regulate the synaptonemal complex (SC) formation, thus explaining the shortened AEs and abnormal synapsis we observed in *Rbm46* KO animals. Our study uncovers a post-transcriptional role for RBM46 in meiosis and characterizes RBM46 as a regulator of both male and female fertility.

## Results

### RBM46 is a germ-cell-specific cytoplasmic RBP required for gametogenesis

**Figure 1.**
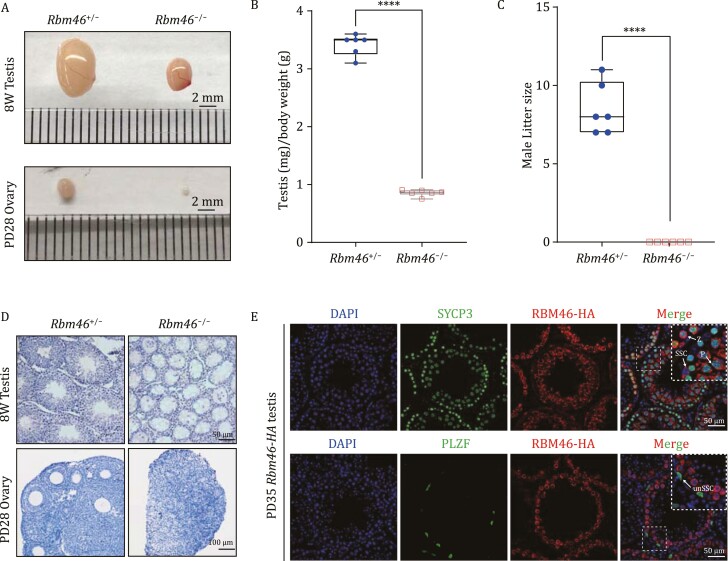
Germ-cell-specific RBM46 is required for male and female fertility. (A) Morphological analysis of testes and ovaries showed that the *Rbm46*^−/−^ mice testes and ovaries were smaller than respective littermate controls (*Rbm46*^+/−^). The scale bar is 2 mm. (B) The scatter plot showed the testis/body weight from 8-week-old *Rbm46*^−/−^ mice compared with their littermate controls (*n* = 6). Student’s *t*-tests were performed, ****P < 0.0001. (C) Number of pups per litter from male mice (>8 weeks old) naturally crossed with WT female mice for 6 months (*n* = 6). *Rbm46*^−/−^ male mice showed complete sterility. Student’s *t*-tests were performed, ****P < 0.0001. (D) Hematoxylin and eosin (H&E) staining of testes and ovaries in control and *Rbm46*^−/−^ mice. Spermatids were absent in the seminiferous tubules of the adult *Rbm46*^−/−^ mice and only a few spermatocytes were present. Oocytes and follicle structures were completely absent in the 1-month-old mice ovaries. (E) Immunofluorescence costaining RBM46-HA-tag (red) with the AR marker SYCP3 and undifferentiated SSC marker PLZF showing that RBM46 localization in the cytoplasm of germ cells in testis sections of RBM46-HA-tag mice. White arrows indicate germ cell stages: Z, zygotene spermatocytes; P, pachytene spermatocytes; unSSC, undifferentiated SSCs. The scale bar is 50 µm.

We used CRISPR/Cas9 to generate *Rbm46* knockout (*Rbm46*^−/−^) mice, and immunoblotting of *Rbm46*^−/−^ testes confirmed successful knockout (Fig. S1B). Upon examining reproductive organs, it was immediately obvious that the *Rbm46*^−/−^ mice testes and ovaries were smaller than these organs in their respective littermate controls (*Rbm46*^+/−^), and the *Rbm46*^−/−^ testes and ovaries weights were significantly lower than the *Rbm46*^+/−^ organs (Fig. 1A and 1B). Consistent with these striking reproductive morphologies, fertility testing showed that neither male nor female *Rbm46*^−/−^ mice were fertile (Figs. 1C and S3B). Further, histological analysis showed that the *Rbm46*^−/−^ ovaries lacked oocytes and that the *Rbm46*^−/−^ testes lacked spermatids in seminiferous tubules (Fig. 1D). These results support that RBM46 is essential for normal reproductive development and that RBM46 is required for both male and female fertility.

Immunoblotting of extracts from major organs of wild-type C57BL/6 mice supported the reproductive-organ-specific accumulation of the RBM46 protein. We also examined RBM46 in testes samples from postnatal day 3 up to 12 weeks, which revealed strong accumulation starting from postnatal day 8 (PD8), the developmental point at which spermatogonial stem cells (SSCs) begin to differentiate (Bellve *et al.*, 1977). Note that we also detected RBM46 accumulation in wild-type E17.5 mice ovaries (Fig. S1B). These findings support that RBM46 is a reproductive-organ-specific protein that apparently functions in meiotic I. To profile the spatiotemporal expression and subcellular localization of RBM46 we used CRISPR/Cas9 to generate mice harboring an HA-tag fusion variant of RBM46. Immunofluorescence staining against the HA-tag, the meiotic AE marker SYCP3, and the undifferentiated SSC marker PLZF showed that RBM46 is a cytoplasmic RBP with weak accumulation in SSCs, with high accumulation in meiotic I spermatocytes, and with no detectable expression in spermatids (Fig. 1E).

### The germ cells from *Rbm46*-null mice fail to progress through meiosis I

**Figure 2.**
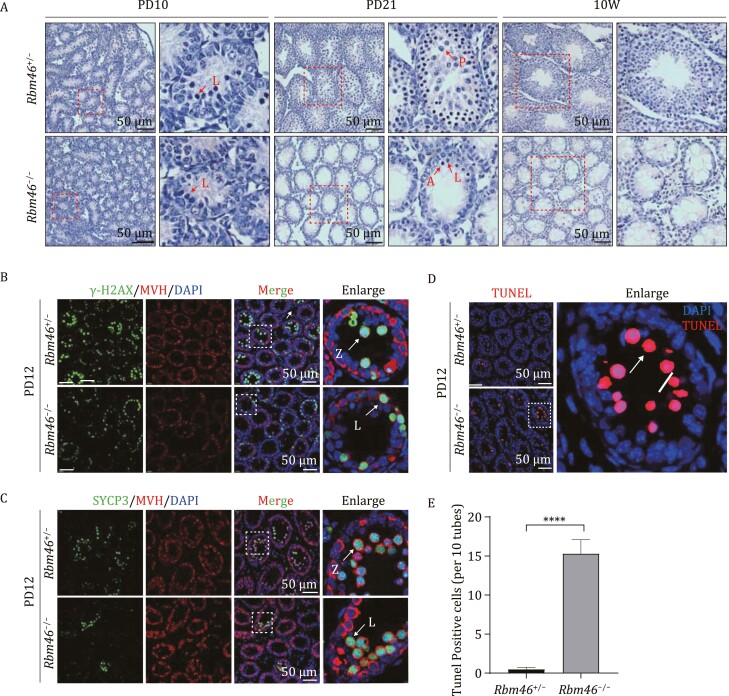
RBM46 deletion causes meiosis arrest at the leptotene stage. (A) H&E staining of control and *Rbm46*^−/−^ mice testes at PD10 (postnatal day 10), PD21, and at 10 weeks, showing inhibition of meiosis in *Rbm46*^−/−^ testes. Red arrows indicate the representative stages of the spermatocytes, L, leptotene spermatocytes; P, pachytene spermatocytes; A, apoptotic cell. The scale bars are shown in the image. (B) Immunofluorescence staining for the DSB marker γ-H2AX (green) and the germ cell marker MVH (red) in controls (*Rbm46*^+/−^) and *Rbm46*^−/−^ testes at PD12. Insets show an enlarged version of one seminiferous tubule. L, leptotene spermatocytes; Z, zygotene spermatocytes. The scale bar is 50 µm. (C) Immunofluorescence staining for the AE marker SYCP3 (green) and the MVH (red) in controls (*Rbm46*^+/−^) and *Rbm46*^−/−^ testes at PD12. Insets show an enlarged version of one seminiferous tubule. The scale bar is 50 µm. (D) Tunnel staining showed that there were more apoptotic cells in the seminiferous tubule of *Rbm46*^−/−^ testis than in control testis; the enlarged region highlights that all of the apoptotic cells are apparently leptotene or zygotene spermatocytes. Arrow, an apoptotic cell. (E) Quantification of the number of apoptotic cells in seminiferous tubules in PD12 testes. Ten adjacent tubules were examined as one group, and three testis sections were examined from control and *Rbm46*^−/−^ mice. Student’s *t*-tests were performed, ****P < 0.0001.

Given the observed fertility and spermatogenesis defects of the *Rbm46*^−/−^ mice, we were interested in determining when the apparent gametogenesis dysfunction starts. Mice begin producing spermatocytes around postnatal day 10 (Feng *et al.*, 2014); we sampled *Rbm46*^+/−^ and *Rbm46*^−/−^ testes at time points including PD8, PD10, PD12, PD21, and PD70 (10 weeks) and conducted histological analysis. We did not observe any obvious between *Rbm46*^−/−^ testes and *Rbm46*^+/−^ testes in PD8 mice. Defects were evident throughout this time course, starting with an obviously reduced number of spermatocytes entering meiosis in PD10 *Rbm46*^−/−^ testes. And the reduction of meiotic spermatocytes became more severe in PD12 *Rbm46*^−/−^ testes. At PD21, *Rbm46*^+/−^ testes had normal round spermatids, whereas no round spermatids were observed in the *Rbm46*^−/−^ mice. And these changes were even more pronounced in the adult (PD70) testes (Figs. 2A and S2A).

We also conducted histological analysis of *Rbm46*^+/−^ and *Rbm46*^−/−^ ovaries sampled across a developmental series and found that *Rbm46*^+/−^ ovaries underwent normal development, whereas few meiotic cells were present in E17.5 *Rbm46*^−/−^ ovaries, and no oocytes were observed in postnatal mice ovaries (Fig. S3A). To observe the potential effect(s) of RBM46 on SSC maintenance and/or differentiation we performed immunofluorescence staining of PD6 and PD10 testes against the undifferentiated SSC marker PLZF and the differentiated SSCs marker LIN28. The intensity of the PLZF signal did not change between *Rbm46*^+/−^ and *Rbm46*^−/−^ testes at PD6 and PD10, whereas the LIN28 signal was slightly decreased in PD10 *Rbm46*^*−*/−^ testes (Figs. S4A and 4B). These results indicate that RBM46 has a slight effect on differentiated SSCs, but no significant effect on the maintenance of undifferentiated SSCs.

We subsequently conducted immunofluorescence staining of seminiferous tubule sections of PD12 testes with the DSB marker γ-H2AX and the AE marker SYCP3. Both markers were detected starting from the early stage of meiosis I (Scherthan *et al.*, 1996; Mahadevaiah *et al.*, 2001), and revealed an obvious reduction in the number meiotic spermatocytes in the *Rbm46*^−/−^ testes compared with the *Rbm46*^+/−^ testes (Fig. 2B and 2C). Additionally, the distribution of SYCP3 in *Rbm46*^−/−^ spermatocytes was diffuse compared with the linear SYCP3 pattern in *Rbm46*^+/−^ spermatocytes (Fig. 2C). An obvious reduction in the number of meiotic oocytes was also observed in a comparison of *Rbm46*^−/−^ and *Rbm46*^+/−^ E17.5 ovaries (Fig. S5A). Consistent with the dearth of spermatocytes in PD12 *Rbm46*^−/−^ testes, TUNEL staining with quantification showed a significant increase in the number of apoptotic cells in the seminiferous tubules of the *Rbm46*^−/−^ mice compared with the *Rbm46*^+/−^ mice (Fig. 2D and 2E). Moreover, staining with the aforementioned γ-H2AX and SYCP3 markers revealed that all of the TUNEL-positive cells were leptotene or zygotene stage spermatocytes (Fig. 2B and 2C). Thus, *Rbm46*^−/−^ gametes fail to progress through meiosis I.

### Profiling RBM46-binding sites by LACE-seq

**Figure 3.**
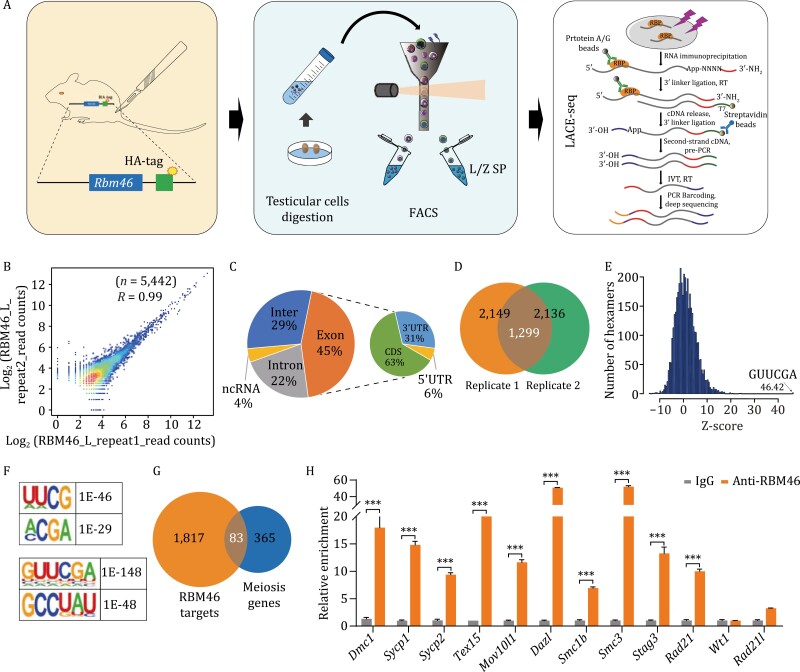
Identification of RBM46 targets of mice testes in L/Z spermatocytes by LACE-seq. (A) Schematic illustration of the experimental workflow. Testes from PD12 RBM46-HA-tagged mice were collected and digested into single cells, after which flow cytometry was used to isolate L- and Z-stage spermatocytes. We subsequently conducted UV crosslinking of these cells and used LACE-seq to identify target RNA molecules (and putative binding sites) of RBM46. L/Z SP, leptotene and zygotene spermatocytes. (B) The LACE-seq was highly correlated in two biological replicates, *R* = 0.99. (C) Genomic distribution of the RBM46 LACE-seq reads in L/Z spermatocytes. Data are plotted using the mean value from two biological replicates. (D) Venn diagram of two biological replicates of RBM46-binding peaks. (E) Histogram showing overrepresented RBM46-binding motifs of identified by LACE-seq using a *K*-mer algorithm. The *Z*-score of the top hexamer is indicated. (F) RBM46-binding motifs identified by LACE-seq using HOMER. (G) 83 genes were identified upon assessing the intersection of candidate LACE-seq target RNA molecules in L/Z spermatocytes and genes with annotated functions in meiosis in the Ingenuity Pathway Analysis (IPA) software. (H) RIP-qPCR confirmed that RBM46 binds to RNA transcripts of 10 putative target mRNAs expressed during meiosis. Among the mRNAs examined the RIP-qPCR data for 10 putative targets showed a >5-fold changed (calculated by 2^−ΔΔCt^) compared with the IgG control, and were therefore considered as “confirmed targets.” In contrast, no enrichment was detected for two nontarget mRNAs that showed a <5-fold changed. Student’s *t*-tests were performed and data are shown as the mean ± SEM of three independent experiments. ***P < 0.001.

Given the predicted function of RBM46 as an RBP (Gerstberger *et al.*, 2014; Table S1), and empowered by very recent methodological progress in the field of RBP target profiling (Su *et al.*, 2021), we pursued the mechanistic basis of the observed meiotic arrest by conducting LACE-seq analysis. Very briefly, this technique exploits an *in vitro* transcription (IVT) approach to amplify trace amounts of cDNAs from single or dozens of cells and can identify the target molecules of RBPs based on ultraviolet (UV) light crosslinking and reverse transcription (RT) of immunopurified protein–RNA complexes (Fig. 3A). We examined leptotene/zygotene spermatocytes isolated by flow cytometry from PD12 testes of the HA-tagged RBM46 mice (i.e., the time point when meiosis I advances to the zygotene stage) (Bellve *et al.*, 1977). The two testes of each mouse were pooled together as one sample, and the single-cell samples isolated by flow cytometry were divided into three groups (each with two biological replicates): an RNA-seq group to establish the background RNA levels, a control group (treated with IgG), and the RBM46 experimental group (treated with HA antibody to pull-down RNAs bound by HA-tagged RBM46).

The two biological replicates of the RBM46 experimental group showed strong correlations in the detected putative binding target RNA molecules (*R* = 0.99) (Fig. 3B). We examined the genomic distribution of the RBM46 LACE-seq reads and found that 45% of the putative binding sites were located in the CDS and 3ʹUTR regions of mRNAs (63% of the RBM46 LACE-seq reads mapped to the CDS; 31% mapped to 3ʹUTR regions) (Fig. 3C). There were a total of 1,299 candidate target RNA molecules common to the two examined replicates (from among 2,149 and 2,136 in each sample) (Fig. 3D and Table S3). Note that this number of target molecules is similar to a previously reported LACE-seq-based analysis of the RBP AGO2 in murine oocytes, and the number of reads for each RBM46 sample (more than 10^7^) is similar to a LACE-seq-based analysis of PTBP1 in HeLa cells (Su *et al.*, 2021).

We employed a *K*-mer algorithm (Berger *et al.*, 2006) to identify the consensus sequence of RBM46-binding sites and discovered the mRNA motifs were enriched in RBM46-binding sites. The top hexamer was GUUCGA, with a *Z*-score of 46.42 (Fig. 3E). We also used HOMER (Heinz *et al.*, 2010) to identify the sequence, and GUUCGA also ranked highest among the most enriched 6-mer motif with a *P* value of 1 × 10^−148^ (Fig. 3F). GCCUAU ranks second with *P* = 1 × 10^−48^. Of particular note, 36.91% of all RBM46 peaks contain a GCCUAU site, and 6.77% of RBM46 peaks contain an GUUCGA site. Among the most enriched 4-mer motifs, UUCG ranked highest with a *P* value of 1 × 10^−46^ (Fig. 3F).

### RBM46-binding targets are enriched for meiosis-related functions

Given our finding that RBM46 deletion results in meiosis arrest at the meiosis I, we focused on meiosis-related molecules among the putative RBM46-target molecules. An analysis using Ingenuity Pathway Analysis (IPA) software (Kramer *et al.*, 2014; Wang *et al.*, 2021) identified 83 molecules with meiosis-related functional annotations among the 1,900 mRNA hits from the LACE-seq analysis for RBM46-binding targets (Fig. 3G). Among these putative targets, we selected 10 molecules for binding validation in PD12 wild-type C57 mouse testes using RNA immunoprecipitation (RIP) assays (Keene *et al.*, 2006); we also measured the binding capacity of two control molecules, a Sertoli cell-specific marker (*Wt1*) and a cohesin subunit (*Rad21l*, i.e., which was not among the putative RBM46-binding targets) (Fig. 3H). All 10 of the putative targets were enriched in the anti-RBM46 immunoprecipitant compared with the IgG control; no enrichment was detected for *Wt1* or *Rad21l*. These findings support that RBM46 interacts *in vivo* with the meiosis-related mRNAs identified in the LACE-seq analysis.

### RBM46 binds at the 3ʹUTR of mRNAs encoding multiple subunits of meiotic cohesin complexes and regulates their translation

Cohesin complexes form ring structures to hold sister chromatids together during mitosis and meiosis, and studies have shown that abnormal formation of cohesin complexes can block meiosis I (from leptotene to pachytene) (Klein *et al.*, 1999; Haering and Jessberger, 2012; Biswas *et al.*, 2016). Among the validated RMB46-binding targets were mRNAs encoding four known cohesin subunits that were enriched in an IPA analysis of Canonical Pathways (Fig. 3H and Table S4). Two of these are meiosis-specific cohesin subunits (*Smc1b* and *Stag3*), while the other two are subunits of ubiquitous cohesin complexes (*Rad21* and *Smc3*). Previous studies have shown that the absence of the meiosis-specific cohesin subunit *Smc1b* results in meiotic arrest in pachytene (Revenkova *et al.*, 2001) and that the absence of *Stag3* results in meiotic arrest in leptotene (Prieto *et al.*, 2001).

**Figure 4.**
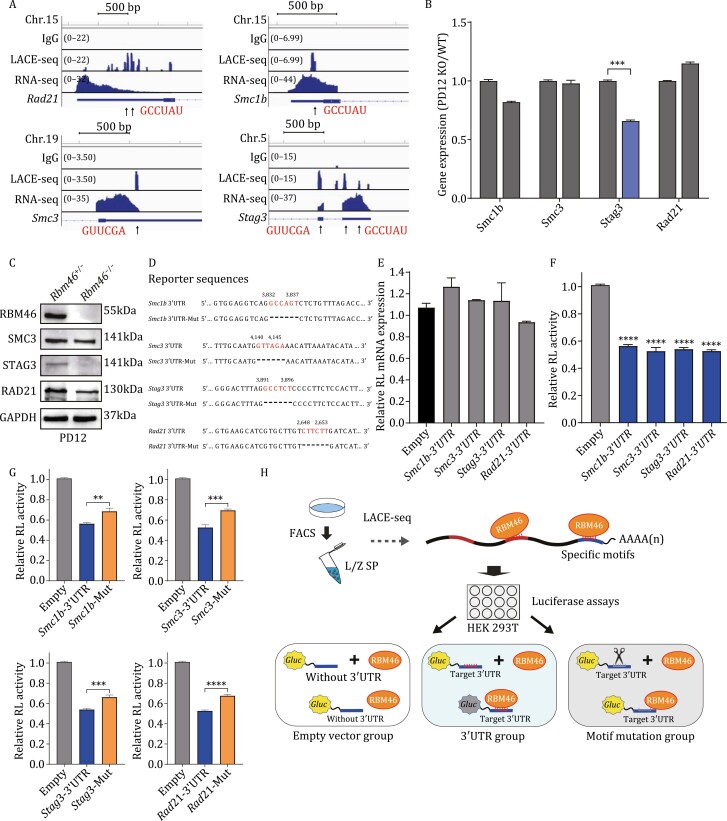
RBM46 binds at specific motifs in the 3ʹUTRs of mRNAs encoding multiple subunits of meiotic cohesin complexes and regulates their translation. (A) Genome browser tracks showing LACE-seq-binding peak distributions and transcript levels of the four meiotic cohesin mRNAs encoded by the mouse genome. Predicted RBM46-binding sites are present in the 3ʹUTRs of *Rad21*, *Smc3*, and *Smc1b*; *Stage3* have binding sites both in 3ʹUTRs and CDS region. (B) qPCR to measure the expression of the four indicated genes encoding meiotic cohesin complex subunits in PD12 *Rbm46*^−/−^mice and in their respective littermate *Rbm46*^+/−^ controls. Data are shown as the mean ± SEM of three independent experiments. *P < 0.05, **P < 0.01, ***P < 0.001; Student’s *t*-tests. (C) Immunoblotting showing that the protein levels of the three indicated meiotic cohesin complex subunits are significantly reduced in PD12 *Rbm46*^−/−^ testes compared with *Rbm46*^+/−^ controls. (D) Predicted RBM46-binding motifs and sequences at the 3ʹUTRs of *Smc1b*, *Smc3*, *Stag3*, and *Rad21* and mutated 3ʹUTR renilla luciferase reporter sequences. (E) qPCR showing that the mRNA levels of luciferase reporter genes were not changed. **P < 0.01, ***P < 0.001, ****P < 0.0001; Student’s *t*-tests. (F) The relative luciferase reporter activity of target cohesin genes showed that RBM46 could specifically bind to their 3ʹUTRs. The luciferase reporter mRNAs containing the 3ʹUTR sequence of target cohesin genes and RBM46 vehicle were cotransfected into the HEK 293 T cells. The renilla luciferase reporter activities were normalized to the SEAP activities. The empty luciferase vehicle cotransfected with RBM46 vehicle served as the control. (G) The decreased luciferase activities were partially rescued in cells cotransfected with the deletion variant control vectors for the *Rad21*, *Smc1b*, *Smc3*, and *Stag3* 3ʹUTRs. (H) Schematic illustration of the luciferase experimental workflow.

We analyzed the LACE-seq-binding peaks and transcript levels of the four RBM46-target cohesin mRNAs (Fig. 4A), and the nontarget cohesin *Rad21l* mRNA as a negative control (Fig. S4A). *Rad21l* had no obvious RBM46-binding peaks; *Stag3* had binding peaks in both its 3ʹUTR and CDS; *Rad21*, *Smc3*, and *Smc1b* had binding peaks in their 3ʹUTRs. Integrative Genomics Viewer (IGV) (Robinson *et al.*, 2011) based analysis of the preferential binding sequences located within the binding peaks identified GCCUAU and GUUCGA as apparent RBM46-binding motifs (Fig. 4A). Our results thus support that RBM46 preferentially interacts with GCCUAU/GUUCGA motifs in the 3ʹUTRs of mRNA encoding cohesin subunits, suggesting that RBM46 functions in regulating sister chromatid cohesion and synapsis.

To investigate the potential post-transcriptional function of RBM46 in regulating targets in mice gametogenesis we examined RBM46-target mRNAs encoding meiotic cohesin complex subunits at both the mRNA and protein levels. qPCR analysis of PD12 testes (when meiosis advances to the zygotene stage) from *Rbm46*^+/−^ and *Rbm46*^−/−^ mice detected no differences in the levels of mRNA transcripts for *Smc1b*, *Smc3*, or *Rad21*, while *Stag3* mRNA levels in *Rbm46*^−/−^ testes displayed a significant reduction (Fig. 4B). Immunoblotting revealed obvious differences between the *Rbm46*^+/−^ and *Rbm46*^−/−^ PD12 testes: the protein levels of SMC3, RAD21, and STAG3 were decreased in the RBM46 KO samples (Fig. 4C). These results indicate RBM46 is a positive regulator of mRNA translation and that deletion of RBM46 in mice testes results in a decrease in the protein levels of its mRNA targets.

### RBM46 binds cohesin subunit mRNAs at the 3ʹUTR via GCCUAU/GUUCGA motifs

Recalling the predicted RBM46-binding motifs in the cohesin subunits mRNAs (Fig. 4A), we conducted dual-luciferase reporter assays to help delineate the regulatory impacts of RBM46 for these specific mRNAs. Dual-luciferase assays were conducted to assess the RBM46 motifs *in vitro*; we inserted the cloned 3ʹUTR regions containing the RBM46-binding motifs into the MCS2 region of the pEZX-GA02 vector (downstream of the sequence encoding a luciferase reporter). These assays examined 3ʹUTR fragments of four cohesin subunit mRNAs, including *Smc3* and *Rad21* (containing GUUCGA motifs) and *Smc1b* and *Stag3* (GCCUAU motifs) (Fig. 4D); we also prepared deletion variant control plasmids for each examined 3ʹUTR fragment (e.g., “*Smc1b* 3ʹUTR-Mut”). HEK 293T cells were then cotransfected with an RBM46 overexpression vector and one of the vectors bearing the 3ʹUTR fragment of the cohesin subunit (or an empty vector control).

While we observed no difference in the expression levels of the reporter mRNAs (Fig. 4E), the luciferase activities in the cells with the RBM46-binding motifs were reduced by between 50% and 60% compared with the empty vector control cells (Fig. 4F). Moreover, the decreased luciferase activities were partially rescued in cells cotransfected with the deletion variant control vectors for the *Rad21*, *Smc1b*, *Smc3*, and *Stag3* 3ʹUTRs (Fig. 4G). These findings demonstrate that RBM46 binds to GCCUAU/GUUCGA motifs in 3ʹUTRs *in vivo* and indicate that such binding disrupts the translation of mRNA transcripts bearing these motifs (Fig. 4H).

### *Rbm46* ^−/−^ arrested spermatocytes have shortened AEs and display partial synapsis

We next investigated how loss of RBM46 results in spermatocyte arrest during meiosis I. We narrowed our focus to specific meiotic stages in chromosome spreads prepared from PD35 *Rbm46*^+/−^ and *Rbm46*^−/−^ mice testes using immunofluorescence staining against SYCP1 and SYCP3. For context, cohesin complexes together with other axial proteins such as the SC protein SYCP3 form the two lateral elements of the SC (also referred to as AEs upon completion of synapsis); these are bridged together by the central element protein, SYCP1, and this bridging is known to stimulate synapsis of homologous chromosomes (Meuwissen *et al.*, 1992; Yuan *et al.*, 2000). SYCP3 is expressed starting from the preleptotene stage (Scherthan *et al.*, 1996), while SYCP1 marks synapsed chromosomes and are expressed starting from the early zygotene stage (de Vries *et al.*, 2005).

**Figure 5.**
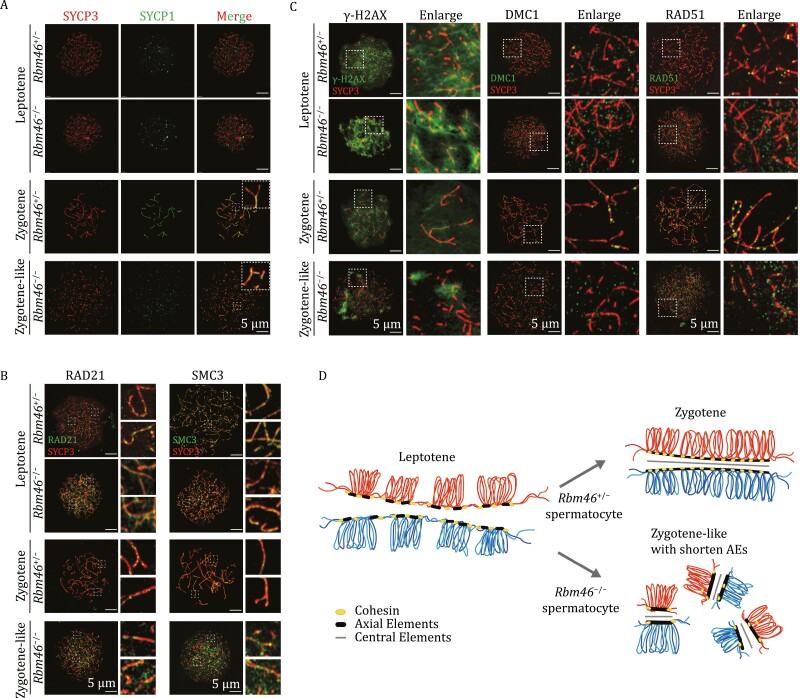
*Rbm46* ^−/−^ arrested spermatocytes have shortened AEs and display partial synapsis. (A) Chromosome spreads of PD35 *Rbm46*^+/−^ and *Rbm46*^−/−^ spermatocytes costained with the chromatid central element marker SYCP1 (green) and the AE marker SYCP3 (red), showing that *Rbm46*^−/−^ spermatocytes arrest at the leptotene stage and present “zygotene-like” spermatocytes. The scale bar is 5 µm. (B) Chromosome spread of *Rbm46*^+/−^ and *Rbm46*^−/−^ mice spermatocytes costained with SYCP3 (red) and cohesin subunits RAD21 and SMC3 in leptotene and zygotene or zygotene-like spermatocytes. The scale bar is 5 µm. (C) Chromosome spread of *Rbm46*^+/−^ and *Rbm46*^−/−^ mice spermatocytes costained with SYCP3 (red) and γ-H2AX as indicated for DSB signal; DMC1 and RAD51 as indicated for DSB-repair foci. The scale bar is 5 µm. (D) Model of RBM46 functional mechanism in meiosis. Abnormal distribution of cohesin subunit proteins was observed in *Rbm46*^−/−^ mice spermatocytes, and chromosomes showed shortened AEs and partial synapsis, resulting in failure of meiosis to progress to the zygotene stage.

A previous study conducted costaining of *Rbm46*^+/−^ spermatocyte chromosome spreads and observed that chromosome axes were positive for both SYCP1 and SYCP3 in zygotene spermatocytes, whereas leptotene spermatocytes were only positive for SYCP3 (Meuwissen *et al.*, 1992; Cahoon and Hawley, 2016). In *Rbm46*^−/−^ mouse spermatocytes, we observed leptotene spermatocytes with the expected SYCP3 localization, but no spermatocytes developed beyond the zygotene stage (Fig. 5A). Note that we also observed that oocytes arrest at leptotene stage in E17.5 *Rbm46*^−/−^ mice ovaries (Fig. S5A). We did observe many abnormal spermatocytes displaying shortened chromosome axes (indicated by a fragmented SYCP3 signal), and some of these abnormal spermatocytes had a signal for SYCP1 (Fig. 5A). We refer to these spermatocytes present in the *Rbm46*^−/−^ testes as “zygotene-like” spermatocytes. Specifically, the presence of these spermatocytes indicates that a partially synapsed, SC-like structure was initiated, but failed to progress normally toward the zygotene stage.

Next, we observed changes in RBM46-target cohesin subunits during meiosis by costaining RAD21 and SMC3 with SYCP3 in *Rbm46*^−/−^ and *Rbm46*^+/−^ spermatocytes (Fig. 5B). Compared with the *Rbm46*^+/−^ chromosomal uniform point distribution, RAD21 was not clearly clustered, but rather diffused around chromosomes in both *Rbm46*^−/−^ leptotene and zygotene-like spermatocytes. The localization of SMC3 on chromosomes did not differ significantly between *Rbm46*^−/−^ and *Rbm46*^+/−^ leptotene, but the loci signal appears to be partially localized on chromosomes in zygotene-like spermatocytes of *Rbm46*^−/−^ mice (Fig. 5B). These results suggest that deletion of *Rbm46* disrupts the loading of cohesin subunits onto chromosomes.

Meiotic recombination is initiated by programmed DSBs (Siegel *et al.*, 1992). We then examined the effect of *Rbm46* deletion on DSB processes, as cohesin plays an important role in DSB repair. We costained PD35 *Rbm46*^+/−^ and *Rbm46*^−/−^ mice testes against SYCP3 and the unsynapsed chromosome region marker γ-H2AX (Fig. 5C). All of the *Rbm46*^−/−^ spermatocytes were positive for γ-H2AX staining at leptotene, and the γ-H2AX signal intensity was partially reduced in the zygotene-like spermatocytes. We also stained against the DSB-repair proteins DMC1 and RAD51 and found no differences in signal intensities between *Rbm46*^−/−^ zygotene-like and *Rbm46*^+/−^ zygotene spermatocytes (Fig. 5C). However, without normal AEs in *Rbm46*^−/−^ zygotene-like spermatocytes, accumulation of foci on axes was obviously reduced, and the DSBs were not processed normally (as the homologous partner chromosome was not available owing to incomplete synapsis of the two homologous pairs of sister chromatids). These results indicate that although DSB formation proceeds normally in *Rbm46*^−/−^ testes, the formed DSBs cannot be repaired normally owing to the failure of synapsed chromosome formation.

## Discussion

We generated *Rbm46* knockout mice and observed their spermatocytes arrest in early meiosis. And found that RBM46 KO spermatocytes cannot synapse properly and fail to progress toward the zygotene. To investigate the mechanism(s) through which RBM46 KO causes meiotic arrest, we sorted wild-type L/Z spermatocytes by flow cytometry and used LACE-seq to profile RNA-binding targets of RBM46. Analysis of the LACE-seq-binding peaks showed that RBM46 binds to specific motifs (GCCUAU/ GUUCGA) in the 3ʹUTRs of mRNAs for cohesin subunits including *Rad21*, *Smc3*, *Smc1b*, and *Stag3*. The knockout of *Rbm46* disrupts the assembly of axil elements and of synapses by reducing the protein translation of cohesin subunits.

RBPs are commonly divided into nuclear RBPs and cytoplasmic RBPs: nuclear RBPs are known to function in (e.g.) regulating nascent mRNA (pre-mRNA) and various processing events including capping, polyadenylation, and splicing; cytoplasmic RBPs often bind mature mRNA sequences as they are released from the nucleus, and have been shown to function in directing mRNA transport, regulating interactions with translation machinery (competitively or cooperatively), and regulating mRNA stability (Sutherland *et al.*, 2015).

Numerous studies have reported that cytoplasmic RBPs can regulate mRNAs stability and translation by binding to the 3ʹUTR regions of target mRNAs. For example, NANOS2 is an RBP essential for the maintenance of SSCs through the regulation of target mRNAs stability. Using the CRAC technique, 81% of NANOS2-binding sites were identified as located in the 3ʹUTR of transcripts by a consensus motif AUKAAWU (K = G or U, W = A or U) (Codino *et al.*, 2021). LIN28A is a positive regulator of mRNA translation in various cells (Polesskaya *et al.*, 2007; Jin *et al.*, 2011). HITS-CLIP experiments showed that LIN28A preferentially binds to the 3ʹUTR regions of several meiosis-associated mRNAs, and dual-luciferase assays confirmed that LIN28A binds a GGAG(A) motif in the 3ʹUTRs to regulate its target mRNAs (Wang *et al.*, 2020a).

In cynomolgus monkey ovaries, RBM46 was observed as localized to the oocyte cytoplasm (Wang *et al.*, 2020b). Our data for the subcellular localization of RBM46 in mouse testes indicate that RBM46 is localized in the cytoplasm of SSCs and meiotic I spermatocytes. Given this localization pattern, it is reasonable to infer that RBM46 may affect the translation of its binding target mRNA transcripts and/or may regulate their mRNA stability. In our study, two hexamer motifs (GUUCGA and GCCUAU) were identified as enriched at RBM46-binding sites, and dual-luciferase assays experimentally confirmed the specificity of RBM46 binding and demonstrated that RBM46 binding at these 3ʹUTR sites promotes the translation of bound mRNA molecules.

The functions of multiple subunits of the meiosis-specific cohesin complex have been investigated in knockout mice. *Smc1b*-null mice showed a phenotype of shortened chromosome axes and incomplete synapses, and also displayed aberrant telomere integrity (Revenkova *et al.*, 2001; Adelfalk *et al.*, 2009). *Rec8*-null mice also displayed shortened chromosome axes in meiotic cells, and had defects in sister SC assembly and repair of DSB (Lee *et al.*, 2003; Xu *et al.*, 2005). *Rad21L*-null mice displayed fragmented chromosome axes, nonhomologous synapsis, and impaired DSB repair (Herran *et al.*, 2011; Lee and Hirano, 2011). *Stag3*-null mice displayed chromosome axis compaction, aberrant synapsis, impaired recombination, and developmental arrest (Prieto *et al.*, 2001; Fukuda *et al.*, 2014). Notably, *Rec8* and *Rad21L* double-null mice exhibited a more severe phenotype than the corresponding single mutants, failing to form meiotic chromosome axes and failing to assemble AEs or SCs (Llano *et al.*, 2012). Another study reported that double mutants of *Stag3* with either *Rec8* or *Rad21L* mice displayed defective centromeric cohesion and very short AEs, similar phenotypes to the *Rec8* and *Rad21L* double-null mice (Ward *et al.*, 2016).

In the present study, we observed that protein levels of cohesin subunits SMC3, STAG3, and RAD21 were decreased in PD12 *Rbm46*^−/−^ testes. Chromosome spreads indicated that the localization of RAD21 and SMC3 in *Rbm46*^−/−^ spermatocytes was diffuse as compared with the foci signal observed in *Rbm46*^+/−^. Knockout of RBM46 disrupts the assembly of SCs due to shortened AEs compared with normal chromosomes, which is similar to the reported phenotypes in *Stag3* and *Rad21L* null mice (Jessberger, 2011; Fukuda *et al.*, 2014). The knockout of *Rbm46* reduces the translation of cohesin subunits and disrupts the assembly of AEs and synapses; these collectively result in the failure of meiosis to progress normally to the zygotene stage. Interestingly, unlike the other three cohesin subunits, we observed a decrease in *Stag3* mRNA expression. It is known that the regulation of spermatogenesis by RBPs is complex (Legrand and Hobbs, 2018). The decreased expression of *Stag3* may be due to the multiple binding sites of RBM46 on *Stag3*, including CDS and 3ʹUTRs regions. It is also possible that RBM46 interacts with other molecules and affects the transcription of *Stag3*, which needs further study.

It is clear that some tissues tend to be enriched for specialized RBPs: 90% of the 82 tissue-specific RBPs detected to date in human were identified specifically in the germline, brain, muscle, bone marrow, or liver cells. Of particular note, 57% of these (47 proteins) show enrichment in adult testis, where they have been variously shown to function in gametogenesis and fertility by regulating meiosis, stem cell maintenance, and differentiation (Kang and Han, 2011; Luteijn and Ketting, 2013; Gerstberger *et al.*, 2014).

Characterization studies using transgenic models of reproductive system-expressed RBPs disrupted commonly present an infertile phenotype with spermatogenesis arrest. For example, the germline-specific RBPs DAZ and BOULE are required for male fertility (Reijo *et al.*, 1995; VanGompel and Xu, 2010), while DAZL is required for both male and female fertility, and is known to influence both embryonic germ cell development and differentiation. During spermatogenesis, DAZL binds to at least 1,325 testicular mRNA transcripts at their 3ʹUTR regions (Li *et al.*, 2019); this binding stimulates their translation by recruiting PABPs to these target mRNAs. Notably, DAZL binding had no obvious effects on the cellular levels of the mRNA transcripts *per se* (Li *et al.*, 2019). Additional RBPs are also known to function in spermatogenesis, including DDX5, which exerts multiple functions in RNA splicing, export, and stability and is essential for the maintenance of spermatogenesis (Legrand *et al.*, 2019). The RBM family protein RBM5 was identified as a male germ cell splicing factor required for spermatid differentiation, and knockout of RBM5 results in spermatogenesis arrest at the spermatid stage (O’Bryan *et al.*, 2013). An infertility phenotype was reported for zebrafish lacking RBM46; RBM46 KO animals failed to initiate meiosis (Dai *et al.*, 2021). In the present study, we found that both male and female *Rbm46*^−/−^ mice were infertile and observed arrested meiosis in spermatocytes/oocytes at the leptotene stage.

RNA immunoprecipitation with sequencing (RIP-seq) and crosslinking immunoprecipitation coupled with high-throughput sequencing (CLIP-seq or HITS-CLIP) are two experimental approaches for identifying RBP target molecules (Licatalosi *et al.*, 2008; Zhao *et al.*, 2010). Several related methods have been developed seeking to reduce the required amount of input cells, such as tRIP-seq and TRIBE (McMahon *et al.*, 2016; Masuda *et al.*, 2020). When studying reproductive development, it is difficult to collect a large amount of cell materials at specific stages. To date, most target profiling studies of reproductive system-expressed RBPs have employed CLIP-seq. In a DAZL target profiling study, 16 testes were pooled together as one sample for identifying DAZL targets during spermatogenesis (Li *et al.*, 2019); however, heterogeneity in the sampled developmental stages of the targeted cells (germ cells) and inclusion of other cell types (i.e., Sertoli cells, hemocytes) can reduce the accuracy of such results. Another study sought to identify MIWI targets from isolated, stage-specific spermatids using a unit gravity sedimentation method (Gou *et al.*, 2014; Zhang *et al.*, 2015). While this method is better than using whole testes, it requires a large number of animals.

In the present study, we used LACE-seq, a method to identify RBP-binding sites in as few as a single cell to accurately study the targeting of RBM46 in leptotene and zygotene spermatocytes. LACE-seq achieves a high repeatability and stability and found that RBM46 can bind to specific motifs (GCCUAU/GUUCGA) in the 3ʹUTRs of target RNA molecules. We believe that LACE-seq opens the door to studying the roles of RBPs in previously uncharted cell type and can expedite the discovery of RNA regulatory networks in various diseased and healthy cells.

## Materials and methods

### Mice generation and maintenance

Genomic DNA fragments covering exons 2–4 were deleted using a CRISPR-Cas9-mediated genome editing system (Cyagen Biosciences, USA) to generate a mouse model of *Rbm46*^−/−^ in a C57BL/6 genetic background. The founders were tested by genotyping with polymerase chain reaction (PCR) and the following DNA sequencing analysis. RBM46-N-HA mice were supplied by Genome Tagging Project (GTP) Center, CEMCS, CAS, which was supported by Shanghai Municipal Commission for Science and Technology Grants (19411951800). The HA protein was coexpressed with RBM46 protein by using CRISPR/Cas9 technology without affecting the expression of RBM46 protein by inserting the HA-tag protein gene sequence in the N terminal of RBM46 protein. Mice housing and all experimental protocols were approved by the Regional Ethics Committee of Shandong University.

### Tissue collection and histological analysis

Testes of mice were removed immediately after euthanasia by dissection, fixed in 4% (mass/volume) paraformaldehyde (Solarbio, Beijing, China, P1110) and embedded in paraffin after dehydration. 5 µm sections were prepared and mounted on glass slides. After degreasing the sections were stained with hematoxylin for histological analysis. The sections were also used for immunofluorescence analysis and TUNEL staining. The sections were dewaxed, rehydrated, and following treated with 10 mmol/L sodium citrate buffer (pH 6.0) for 15 min in boiling water. After cooled to room temperature, the sections were treated with phosphate-buffered saline (PBS) containing 0.1% Triton X-100, and then washed three times with PBS (pH 7.4). After blocking with 5% bovine serum albumin, primary antibodies were added to the sections and incubated overnight at 4°C, followed by incubation with Alexa Fluor 488- or 594-conjugated secondary antibodies (1:500 dilution, Abcam; ab150080, ab150120, ab150117). The primary antibodies for immunofluorescence included Anti-phospho-Histone H2A.X (Ser139) Antibody (1:1,000 dilution; Millipore, 05-636), Anti-DDX4/MVH (1:1,000 dilution; Abcam, ab13840), Anti-GCNA1 antibody (1:200 dilution; Abcam, ab82527), HA-tag (1:500 dilution; Cell Signaling Technology, 3724), and mouse anti-SYCP3 (1:500 dilution; Abcam, ab97672). After being washed with PBS three times, the slides were mounted using Mounting medium with DAPI-aqueous, fluoroshield (Abcam, ab104139). TUNEL staining was performed followed the manufacturer’s instructions (KeyGEN BioTECH, KGA7072).

### Chromosome spread and immunofluorescence analysis

Spermatocyte spreads were prepared as previously described by Peters *et al.* (1997). Primary antibodies used for immunofluorescence were as follows: rabbit anti-SYCP3 (1:500 dilution; Abcam ab15093), mouse anti-SYCP3 (1:500 dilution; Abcam, ab97672), rabbit anti-SYCP1 (C-terminal) (1:2,000 dilution; Abcam, ab15090), Anti-phospho-Histone H2A.X (Ser139) Antibody (1:1,000 dilution; Millipore, 05-636), rabbit anti-DMC1 (1:100 dilution; Santa Cruz Biotechnology, sc-22768), rabbit anti-RAD21(1:100 dilution; Abclonal, A18850), rabbit anti-RAD51 polyclonal antibody (1:200 dilution; Thermo Fisher Scientific, PA5-27195) Primary antibody incubated overnight at four degrees in the refrigerator and were detected with Alexa Fluor 488- or 594-conjugated secondary antibodies for 1 h at room temperature. After being washed with PBS several times, the slides were mounted using Mounting medium with DAPI-aqueous, fluoroshield (Abcam, ab104139).

### Western blot

Tissues were collected to prepare protein extracts from C57BL/6 mice and lysed in TAP lysis buffer containing 50 mmol/L HEPES–KOH, pH 7.5, 100 mmol/L KCl, 2 mmol/L EDTA, 10% glycerol, 0.1% NP-40, 10 mmol/L NaF, 0.25 mmol/L Na_3_VO_4_ and 50 mmol/L β-glycerolphosphate plus protease inhibitors (Roche, 04693132001). After homogenization, the cell extracts were placed on ice stand for 30 min, followed by centrifugation at 4°C at 13,000 ×*g* for 20 min. The supernatant was extracted for immunoblotting assays. Equal amounts of proteins were electrophoretic on a 10% BIS-TRIS protein gel (Invitrogen, NP0315) and the bands were transferred to a polyvinylidene fluoride film (Millipore). The primary antibodies for immunoblotting included RBM46 (1:500 dilution; Diaan Wuhan, customed), RAD21 (1:500 dilution; Abclonal, A18850), and GAPDH (1:5,000 dilution; Abways, AB0037).

### Fluorescence-activated cell sorting of spermatocytes

Fluorescence-activated cell sorting (FACS) purification was conducted as previously described to collect leptotene/zygotene spermatocytes from PD12 HA-RBM46 mice for LACE-seq (Bastos *et al.*, 2005; Gaysinskaya *et al.*, 2014; Lima *et al.*, 2017). After removing the tunica albuginea, the testes were placed in 5 mL PBS containing collagenase type I (120 U/mL) and mixed for 10 min at 35°C. The testes were digested in 5 mL of 0.25% trypsin plus 0.1 mL of deoxyribonuclease I (5 mg/mL) at 35°C for 8 min and then terminated by the addition of 0.5 mL fetal bovine serum. The suspension was passed through a 70 µm honeycomb filter. Centrifuge the cell suspension at 4°C at 500 ×*g* for 5 min and remove the supernatant. And then cells sediment was resuspended in 1 mL Dulbecco’s modified Eagle medium with 40 µg Hoechst 33342, 2 μL Zombie NIR™ dye and 5 μL DNase I. The cell suspension was stirred at 34°C for 20 min, then centrifuged at 500 ×*g* for 5 min at 4°C and resuspended in PBS at a concentration of 10^5^ mL for sorting. Using FACS, populations of fluorescently labeled collection cells were collected according to the sorting channel into 1.5-mL LoBind microcentrifuge tubes (Eppendorf, 022431021) containing 0.5 mL PBS. The cell suspension was centrifuged and some of the supernatant was removed leaving about 10 μL PBS.

### LACE-seq

Crosslinking immunoprecipitation coupled with high-throughput sequencing of FACS-purified spermatocyte populations was performed according to Su *et al.*, (2021). The antibody used for immunoprecipitation was: HA-Tag (C29F4) Rabbit mAb (Cell Signaling Technology, 3724) with 3 µg for each test sample. And the IgG from rabbit was used for negative control.

In brief, spermatogonia cells were sorted as described above and collected in 10 μL PBS in 1.5 mL LoBind microcentrifuge tubes. Then cells were irradiated twice with UV-C light on ice at 400 mJ and stayed for 1 or 2 min between 2 radio actions. And these initial steps of LACE-seq exactly followed the options of Su *et al.* (2021), which are RNA immunoprecipitation and fragmentation, RNA dephosphorylation and 3ʹ linker ligation, RT on beads and first-strand cDNA capture by streptavidin beads. Next the Poly(A) tailing was replaced with a 3ʹ cDNA linker adding. The streptavidin beads with first-strand cDNA were resuspended in 20 μL ligation mixture (4.5 µL water, 2 μL 10× ligation buffer, 2 µL ATP (10 µmol/L, NEB, P0756S), 0.5 μL 3ʹ cDNA linker (1 μmol/L, /5pA/CTCGTATGCCGTCTTCTGCTTG/3*NH*_*2*_/, where 5pA denotes 5ʹ phosphorylated adenosine triphosphate, and 3*NH*_*2*_*NH*_*2*_ indicates 3ʹ-amino, synthesized and HPLC purified by Sangon), 1 μL T4 RNA ligase 1, truncated (NEB, M0204S), 10 μL 50% PEG8000) and transferred to a new PCR tube from 1.5-mL LoBind microcentrifuge tubes. The tubes were incubated at 25°C for 24 h in a ThermoMixer C with intermittent vortexing for 15 s at 1,000 rpm every 3 min.

After that, to generate the dsDNA template for IVT, the DNA was preamplified by first PCR with the following mixture: 12.5 μL cDNA, 0.5 μL primer A (GATCACTAATACGACTCACTATAGG, 10 μmol/L; Sangon), 0.5 μL P7 primer (CAAGCAGAAGACGGCATACGAGAT, 10 μmol/L; Sangon) and 12.5 μL 2× KAPA HiFi HotStart ReadyMix (KAPA Biosystems, KK2601). The PCR program was set as follows: 98°C for 3 min; 98°C for 15 s, 60°C for 20 s, and 72°C for 30 s (14–18 cycles); 72°C for 5 min, then hold at 12°C for long time. The PCR tube was then placed on a magnetic rack for 2 min, and the supernatant was transferred to a new LoBind tube and purified by 46.8 μL Ampure XP beads (1.8:1 ratio; Beckman Coulter, A63881) according to the manufacturer’s instructions. 13 μL Water was added to the LoBind tubes to elute PCR products and then transferred to a new PCR tube.

The subsequent IVT, RNA purification, and RT were also completely performed according to Su *et al.* (2021). Then PCR barcoding was performed with 20 μL cDNA, 1 μL P7 primer, 1 μL P5 index primer (AATGATACGGCGACCACCGAGATCTACAC NNNNNACACTCTTTCCCTACACGACGCTCTTCCGATCT,10 μmol/L; Sangon) and 22 μL 2× KAPA HiFi HotStart ReadyMix. And the PCR program was set as follows: 94°C for 3 min; 94°C for 15 s, 62°C for 30 s, and 72°C for 30 s (8–12 cycles); 72°C for 10 min, then hold at 12°C. PCR products between 130 and 300 bp were extracted by agarose gel electrophoresis with a 2% agarose gel and then purified using a gel extraction kit (Qiagen, 28604). The LACE-seq library was single-end sequenced using Illumina HiSeq 2500 at Novogene. LACE-seq data mapping and identification of peak, cluster, and motif are performed following Su *et al.* (2021).

### RNA extraction, RT-qPCR, and RNA-seq

RNA was isolated from HEK 293T cells and flash frozen or fresh testes of *Rbm46*^−/−^ and *Rbm46*^+/−^ mice using FastPure Cell/Tissue Total RNA Isolation Kit V2 (Vazyme, RC112-01) following manufacturer’s instructions. The total RNA was used for subsequent RT-qPCR analysis. The sequences of the primers used are listed in Table S2. The first-strand cDNA was reverse transcribed from total RNA using HiScript III RT SuperMix for qPCR (+gDNA wiper) (Vazyme, R323-01). QPCR was conducted with the guide of SYBR Green Premix Pro Taq HS qPCR Kit (AG, 11701). *Gapdh* gene (LOC107788267) was used as an internal reference and three technical replicates were performed for each experiment. Finally, relative gene expression values were calculated using the 2^2−ΔΔCt^ method, which was subsequently converted to ploidy changes and plotted.

For RNA sequencing, isolated total RNA was converted into cDNA for RNA sequencing using Illumina Truseq RNA Sample Preparation Kit, followed by rRNA removal and sequenced on an Illumina HiSeq 2500 using 2 × 150 nt sequencing.

### RBP immunoprecipitation

RNA-protein complex was captured for qRT-PCR by immunoprecipitation according to the manufacturer’s instructions (Geneseed, P0101). Briefly, tissues were homogenized in immunoprecipitation lysis buffer with RNasin and protease inhibitor and then divided into two samples for anti-HA-RBM46 and anti-IgG as negative controls. Protein A/G Magnetic Beads were used to capture protein at 4°C overnight. RNA of interest was immunoprecipitated with the beads and further analyzed by qPCR.

### Dual-luciferase reporter assay

Dual-luciferase reporters were conducted with Secrete-Pair Dual Luminescence Assay Kit (GeneCopoeia, LF031). The RBM46-bound fragments of targets and their deletion variant, see as Fig. 5C, were inserted into the pEZX-GA02 vector at MCS2 sites. HEK 293 T cells were plated in 12-well plates. Subsequently, a mixture containing 500 ng of reporter plasmid and RBM46 overexpression plasmid was co-transfected to cells with HP DNA Transfection reagent (Roche, 06366546001). And six technical replicates were performed for each group. 72 h later, cells were collected and luciferase activities were assessed according to the manufacturer’s protocols by Microplate luminometer (Berthold, LB960).

## Supplementary Material

pwac040_suppl_Supplementary_Material_S1

pwac040_suppl_Supplementary_Material_S2

pwac040_suppl_Supplementary_Material_S3

## Data Availability

The generated and analyzed data are available in GEO with accession number GSE201190.
