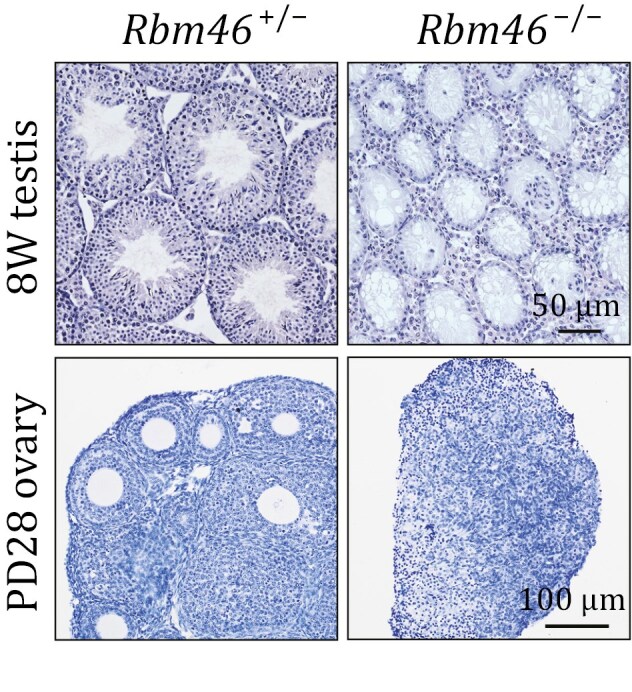# Correction to: RBM46 is essential for gametogenesis and functions in post-transcriptional roles affecting meiotic cohesin subunits

**DOI:** 10.1093/procel/pwaf038

**Published:** 2025-05-20

This is a correction to: Yue Lv, Gang Lu, Yuling Cai, Ruibao Su, Liang Liang, Xin Wang, Wenyu Mu, Xiuqing He, Tao Huang, Jinlong Ma, Yueran Zhao, Zi-Jiang Chen, Yuanchao Xue, Hongbin Liu, Wai-Yee Chan, RBM46 is essential for gametogenesis and functions in post-transcriptional roles affecting meiotic cohesin subunits, *Protein & Cell*, Volume 14, Issue 1, January 2023, Pages 51–63, https://doi.org/10.1093/procel/pwac040.

In the originally published version of this manuscript, there was an incorrect image in Fig. 1, panel D. This should read: