# Supplementary material for: RBM46 is essential for gametogenesis and functions in post-transcriptional roles affecting meiotic cohesin subunits
Source: Protein Cell. 2022 Sep 14;14(1):51–63. doi: 10.1093/procel/pwac040 (PMC9871953; doi:10.1093/procel/pwac040)
Supplement: pwac040_suppl_Supplementary_Material_S1 [file pwac040_suppl_supplementary_material_s1.pdf]

Supplementary Information

RBM46 is essential for gametogenesis and functions in post-transcriptional roles affecting meiotic cohesin subunits

Yue Lv, Gang lu, Yuling Cai, Ruibao Su, Liang Liang, Xin Wang, Wenyu Mu, Xiuqing He, Jin-Long Ma, Yueran Zhao, Zi-Jiang Chen, Yuanchao Xue, Hongbin Liu, Wai-Yee Chan

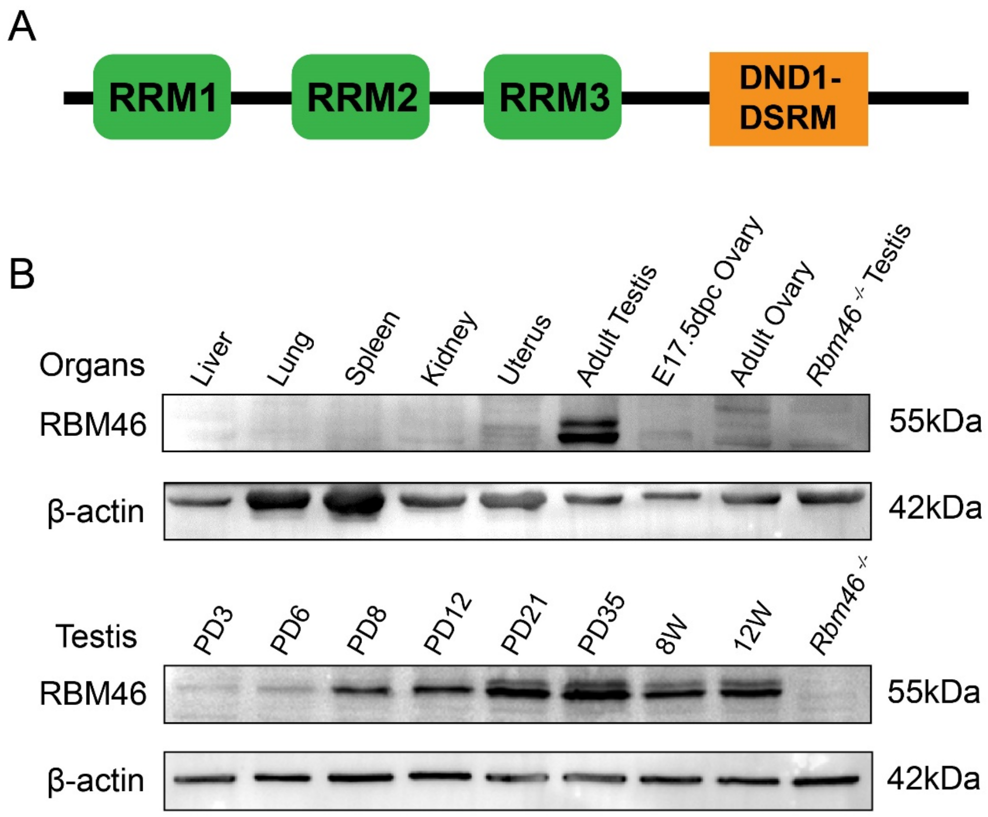

Figure S1. RBM46 domain composition and protein expression profiles in different tissues. (A) Mice RBM46 contains 3 RRM domains and one C-terminal double-stranded RNA binding motif (DSRM) domain. (B) Immunoblotting for the RBM46 protein level in the indicated organs (upper) from wild type mice and testes from *Rbm46* knockout (*Rbm46*$^{-/-}$) adult mice and in a testes age series (down). β-actin was used as the loading control.

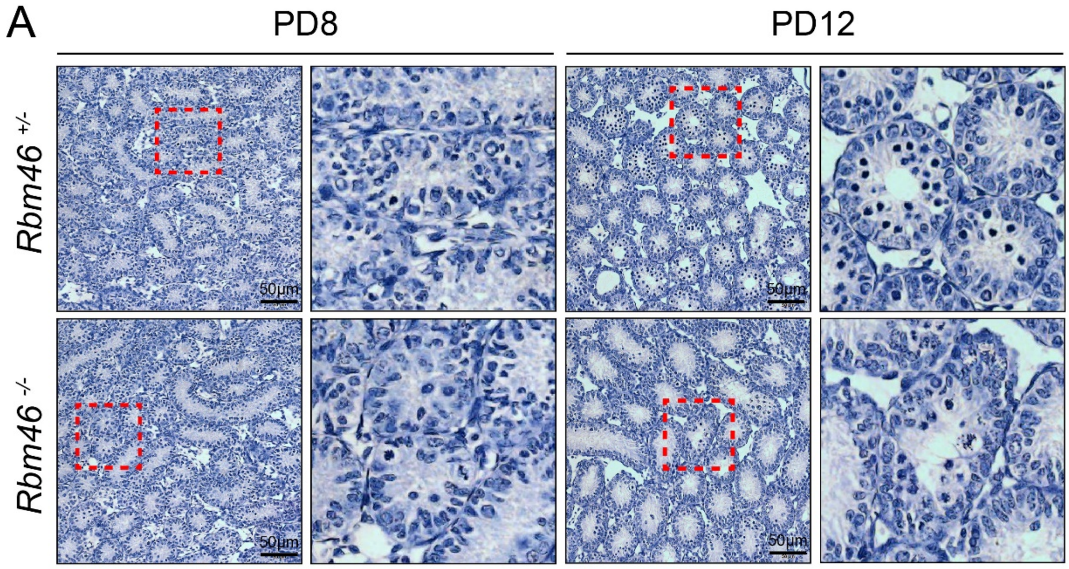

Figure S2. Deletion of *Rbm46* results in a decreased number of meiotic cells in juvenile mouse testes. (A) H&E staining of *Rbm46*$^{+/-}$ and *Rbm46*$^{-/-}$ mice testes at PD8 and PD12. The scale bar is 50 μm.

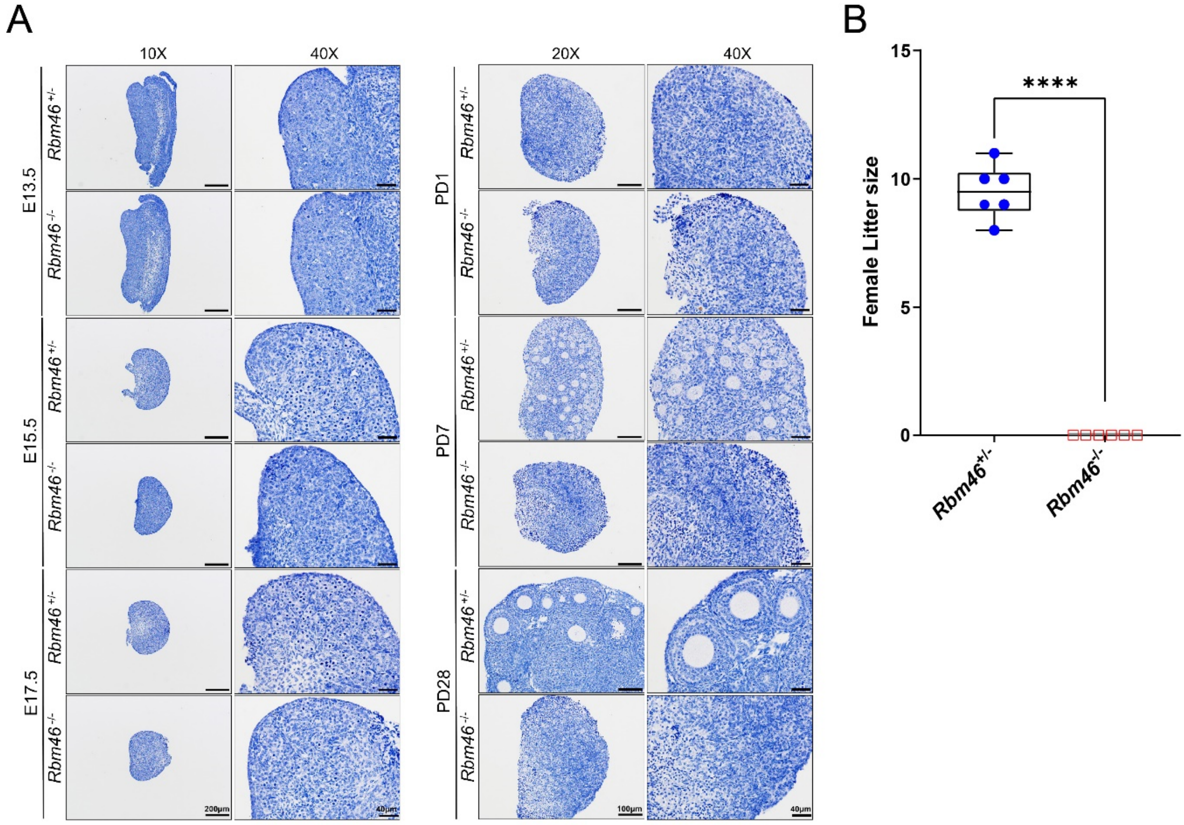

Figure S3. Deletion of *Rbm46* results in the absence of oocytes in female mice after birth. (A) H&E staining of control and *Rbm46*$^{-/-}$ mice ovaries at E13.5 (embryonic day 13.5), E15.5, E17.5, PD1 (postnatal day 10), PD7, and at PD 28, showing inhibition of meiosis and loss of oocytes in *Rbm46*$^{-/-}$ ovaries. The scale bars are shown in the picture. (B) Number of pups per litter from female mice (>8-weeks old) naturally crossed with WT male mice for 6 months (N=6). Student's t tests were performed, **** $P < 0.0001$.

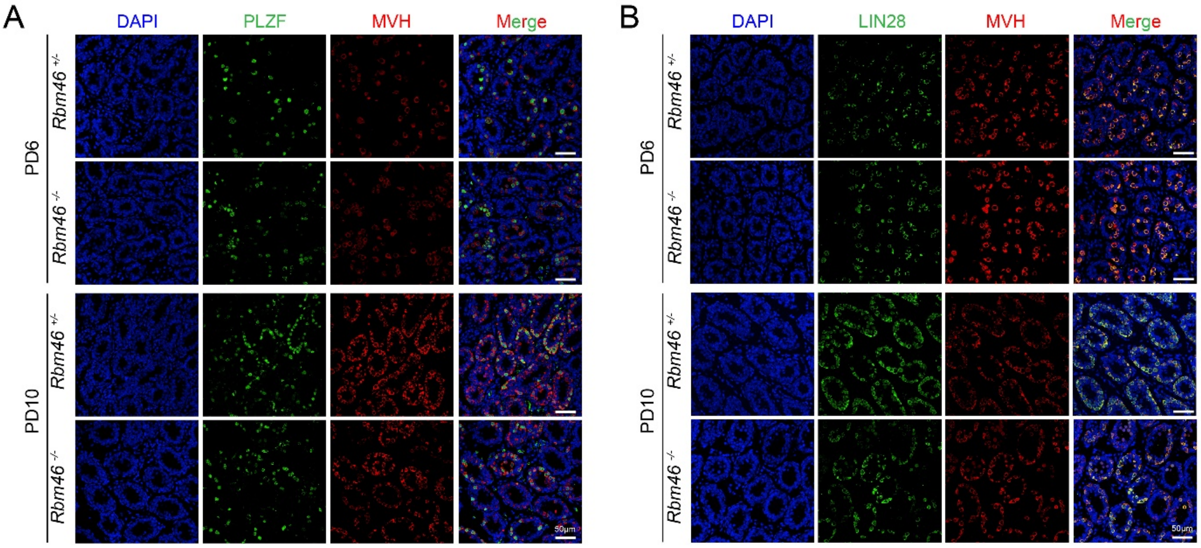

Figure S4. RBM46 Knock out has no significant effect on spermatogonial stem cells. (A) Immunofluorescence staining of *Rbm46*$^{+/-}$ and *Rbm46*$^{-/-}$ testes at PD6 and PD10 against the undifferentiated SSC marker PLZF (green) and the germ cell marker MVH (red) in. (B) Immunofluorescence staining for the differentiated SSCs marker LIN28 (green) and MVH (red) in *Rbm46*$^{+/-}$ and *Rbm46*$^{-/-}$ testes at PD6 and PD10. The scale bar is 50 μm.

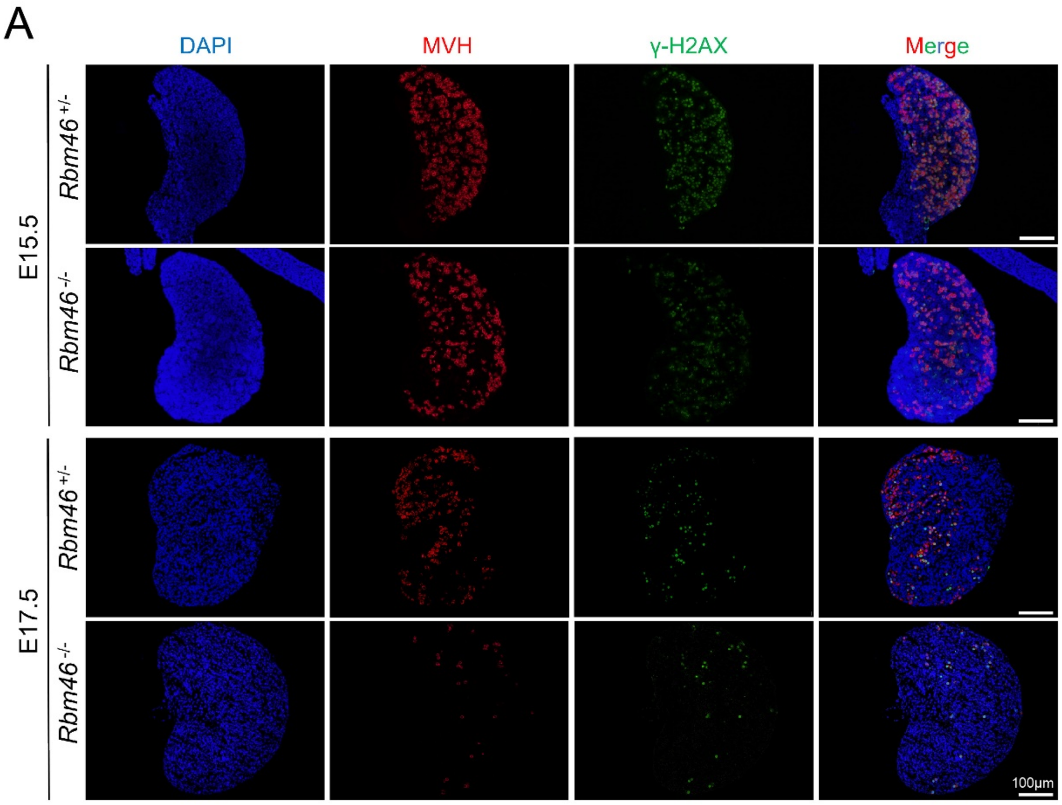

Figure S5. Deletion of *Rbm46* results in a reduction in the number of meiotic oocytes. (A) Immunofluorescence staining for the DSB marker γ-H2AX (green) and the germ cell marker MVH (red) in *Rbm46*$^{+/-}$ and *Rbm46*$^{-/-}$ ovaries at E15.5 and E17.5. The scale bar is 100 μm.

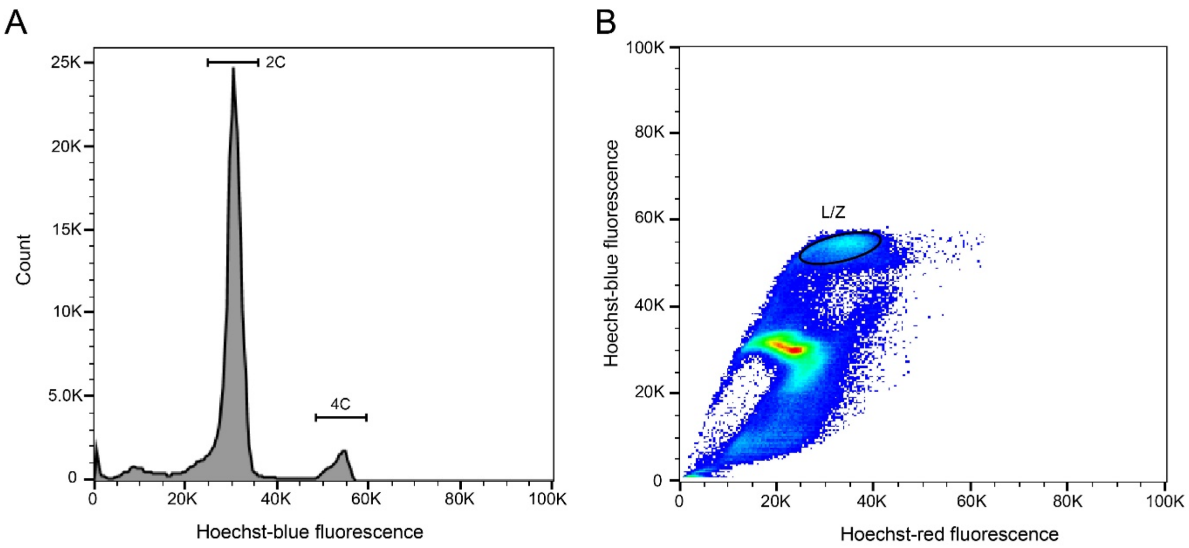

Figure S6. Hoechst-FACS of PD12 HA-RBM46 mice germ cells. (A) Histogram of DNA content ("C") gate; the Y axis represents cell counts and the X axis represents Hoechst blue fluorescence intensity; diploid cells (2C); tetraploid cells (4C). (B) FACS plots of Hoechst 33342 stained testes cells based on Hoechst blue/ red fluorescence. FACS, fluorescence-activated cell sorting, L/Z, leptotene and zygotene spermatocytes.

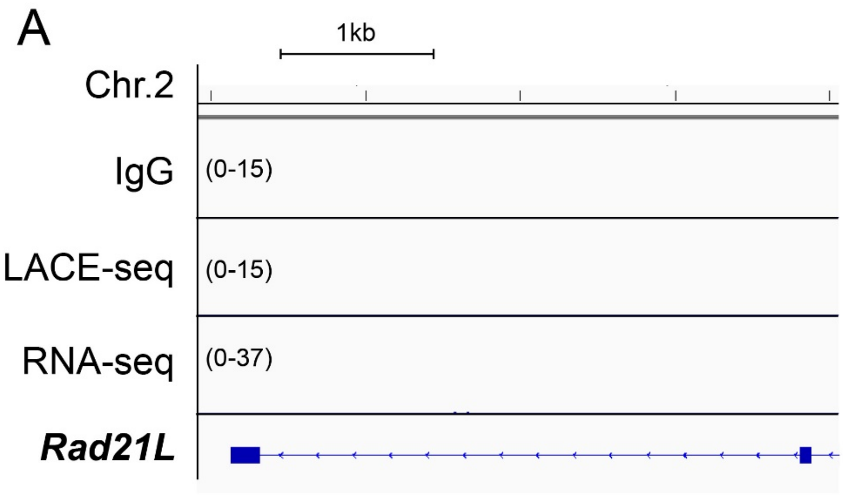

Figure S7. *Rad21l* had no predicted RBM46 binding peaks. (A) Predicted RBM46 binding sites are present in the 3'UTRs of *Rad21l*.

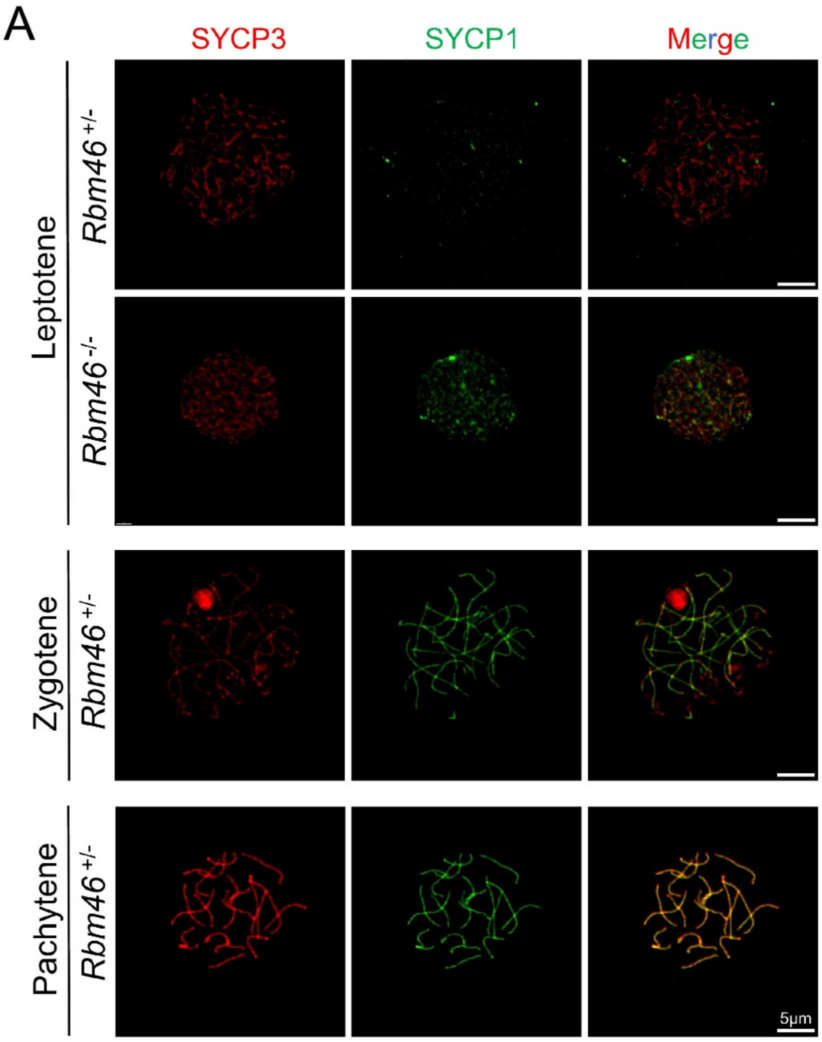

Figure S8. *Rbm46*$^{-/-}$ female mice meiosis arrest at the leptotene stage. (A) Chromosome spread of E17.5 *Rbm46*$^{+/-}$ and *Rbm46*$^{-/-}$ mice oocytes co-stained with chromatid central element marker SYCP1 (green) and axial element marker SYCP3 (red), showing that *Rbm46*$^{-/-}$ oocytes arrest at the leptotene stage and absent normal zygotene oocytes. The scale bar is 5μm.

Table S1. Predictive analysis of DNA/RNA binding proteins of RBM46.

DNA-binding probability	RNA-binding probability
0.1%	100%

Table S2. Primer sequences.

Name	Application (product length)	Forward primer (5'→3')<br>Reverse primer (5'→3')
actb	RT-qPCR (154bp)	GGCTGTATTCCCCTCCATCG
		CCAGTTGGTAACAATGCCATGT
Smc1b	RT-qPCR (187bp)	CAACAGTGCCCAGGCATTTC
		AAAGAATGGAGCAGGCCGAA
Smc3	RT-qPCR (119bp)	CGAAGTTACCGAGACCAAACA
		TCACTGAGAACAAACTGGATTGC
Stag3	RT-qPCR (113bp)	TCCTCAGGCAGTGAGTCTTCC
		GTTCCCTGTGAGTCTCTGTCAT
Rad21	RT-qPCR (106bp)	ATGTTCTACGCACATTTTGTCCT
		TGCACTCAAATACATGGGCTTT
pEZX-GA02	RT-qPCR (254bp)	CAGTGCTGCAATGATACCGC
		GGGAACCGGAGCTGAATGAA
Smc1b-3'UTR	RT-qPCR (162bp)	TCTTGAACAGCCACCAGCTA
		CCATTGGAATGACACTGAGCAC
Smc3-3'UTR	RT-qPCR (319bp)	GCGGCTTTTTGTAAAGTGTCCT
		GGCTAGCCCAAGGAAAGACT
Stag3-3'UTR	RT-qPCR (94bp)	GGACTTTAGGCCTCTCCCCT
		GGCTAGGACCCAAAGAATGC
Rad21-3'UTR	RT-qPCR (309bp)	CCTGCCTAGGAAAATGCCTGA
		GAGGAGTCACGCCACTGTAG
